# Taxogenomic and Metabolic Insights into *Marinobacterium ramblicola* sp. nov., a New Slightly Halophilic Bacterium Isolated from Rambla Salada, Murcia

**DOI:** 10.3390/microorganisms9081654

**Published:** 2021-08-03

**Authors:** Ana Durán-Viseras, David J. Castro, José Carlos Reina, Victoria Béjar, Fernando Martínez-Checa

**Affiliations:** 1 Microbial Exopolysaccharide Research Group, Department of Microbiology, Pharmacy Faculty, Campus de Cartuja s.n., 18071 Granada, Spain; castricoxpc@gmail.com (D.J.C.); josecreina@ugr.es (J.C.R.); vbejar@ugr.es (V.B.); 2 Biomedical Research Center, Institute of Biotechnology, 18016 Granada, Spain

**Keywords:** *Marinobacterium*, hypersaline soil, taxogenomics, metabolism, slightly halophilic

## Abstract

A Gram-negative, motile, rod-shaped bacteria, designated D7^T^, was isolated by using the dilution-to-extinction method, from a soil sample taken from Rambla Salada (Murcia, Spain). Growth of strain D7^T^ was observed at 15–40 °C (optimum, 37 °C), pH 5–9 (optimum, 7) and 0–7.5% (*w*/*v*) NaCl (optimum, 3%). It is facultatively anaerobic. Phylogenetic analysis based on 16S rRNA gene sequence showed it belongs to the genus *Marinobacterium*. The in silico DDH and ANI against closest *Marinobacterium* relatives support its placement as a new species within this genus. The major fatty acids of strain D7^T^ were C_16:0_, summed feature 3 (C_16:1_ *ω*7*c*/C_16:1_ *ω*6*c*) and summed feature 8 (C_18:1_ *ω*7*c*/C_18:1_ *ω*6*c*). The polar lipid profile consists of phosphatidylethanolamine, phosphatidylglycerol and two uncharacterized lipids. Ubiquinone 8 was the unique isoprenoid quinone detected. The DNA G + C content was 59.2 mol%. On the basis of the phylogenetic, phenotypic, chemotaxonomic and genomic characterization, strain D7^T^ (= CECT 9818^T^ = LMG 31312^T^) represents a novel species of the genus *Marinobacterium* for which the name *Marinobacterium ramblicola* sp. nov. is proposed. Genome-based metabolic reconstructions of strain D7^T^ suggested a heterotrophic and chemolitotrophic lifestyle, as well as the capacity to biosynthetize and catabolize compatible solutes, and to degrade hydrocarbon aromatic compounds.

## 1. Introduction

The genus *Marinobacterium* belongs to the family *Alteromonadaceae*, order *Alteromonadales*, class *Gammaproteobacteria*. At the time of writing, the genus *Marinobacterium* comprises 18 different species, from which the type species is *Marinobacterium georgiense*, a later heterotypic synonym of *Marinobacterium iners* [1,2].

Cells of members of this genus are Gram-stain-negative rods, motile by a single or two polar flagella [3]. They are slightly halophilic microorganisms, requiring NaCl for growth [3]. Some members are able to grow on aromatic compounds or to fixate nitrogen under limiting oxygen conditions [3]. Major fatty acids include C_16:0_, C_16:1_ *ω*6*c* and/or C_16:1_ *ω*7*c*, and C_18:1_ *ω*6*c* and/or C_18:1_ *ω*7*c*. Ubiquinone (Q-8) is the major respiratory quinone [3].

Species of the genus *Marinobacterium* have been isolated from a variety of ecological niches including marine habitats, such as tidal flats [4,5,6], marine sediments [7,8,9], seawater [10,11,12,13,14,15], estuarine sediments [16], marine organisms [17], coastal tidal-flat plant rhizospheres [18,19] and pulp mill waste [1]. 

Using culture-independent techniques, members of the genus *Marinobacterium* were also detected in a wide range of habitats including soil samples from tidal freshwater wetlands in China [20], from mangrove river sediment in Taiwan [21], surface sediments from the deep eastern Mediterranean Sea [22] and from the Cochin estuary in the southeastern Arabian Sea [23], seawater samples from the Upper Gulf of Thailand [24], in sodium saccharin wastewater [25], and in different oil or petroleum reservoirs from Brazil, Malaysia and China [26,27,28,29], among others.

During the course of a study of a soil sample collected from Rambla Salada, by using the dilution-to-extinction isolation methodology, strain D7^T^ was isolated in pure culture. This technique allows us to separate individual bacterial cells to initiate enrichment cultures, improving the recovery of slow-growing microorganisms or microorganisms that are apparently uncultivable [30]. 

Rambla Salada is a hypersaline habitat located in the Province of Murcia (southeastern Spain). It was declared as an area with special interest by the European Union, and as a protected wildfowl zone by the regional government of Murcia (BORM 10 September 1998). The salinity of this protected habitat is mainly due to the presence of Na^+^, Cl^−^, SO_4_^2−^ and Ca^2+^ ions. It is an extensive area formed by sedimentary materials in which, due to the low rainfall together with underground water that emerges, originates a number of streams and wadis. The prokaryotic community of Rambla Salada has been studied by our research group during recent decades [30,31,32,33], and we have described so far six novel halophilic bacterial species from this athalassohaline habitat: *Idiomarina ramblicola* [34], *Halomonas cerina* [35], *Halomonas ramblicola* [36], *Blastomonas quesadae* [37], *Roseovarius ramblicola* [38] and more recently, *Roseovarius bejariae* [39].

In the publication at hand, we describe the isolation and taxogenomic characterization of strain D7^T^, based on a polyphasic approach including genomic data. Results confirm it is a new species within the genus *Marinobacterium*, for which we propose the name of *Marinobacterium ramblicola* sp. nov. Moreover, based on a detailed genomic study, the metabolism of this new taxon was studied in depth.

## 2. Materials and Methods

### 2.1. Bacterial Strains

Strain D7^T^ was isolated in this study from a soil sample taken from Rambla Salada, a hypersaline steep-sided river (rambla), located in the Province of Murcia, southeast Spain, 38°07′27.1″ N 1°07′01.4″ W. The physicochemical parameters of pH, oxygen (mg L^−1^) and salinity (g L*^−^*^1^) of the sampling location in Rambla Salada were 6.3, 10.2 and 44.4, respectively.

The sample was collected using a sterile polycarbonate tube, taken immediately to the laboratory and stored at 4 °C until study. The pH of the sample was close to neutral and salinity was around 40 g L^−1^. For the isolation, we used S3 medium, a low-nutrient medium [40] supplemented with 3% (*w*/*v*) sea-salt solution [41] and the dilution-to- extinction approach as a cultivation method, described previously by Castro et al. [38]. This approach is a technique that improves the isolation of slow-growing species or apparently uncultivable species [42,43,44]. The extinction cultures were incubated at 25 °C for 30 days and after, the contents of wells were then re-isolated on Reasoner’s 2A (R2A) medium plates [45]. The isolated strain was maintained and routinely grown in R2A with 3% (*w*/*v*) sea-salt solution at 30 °C as well as on marine agar (MA; 2216, Difco, Sparks, MD, USA). 

For taxonomic comparison purposes, *Marinobacterium zhoushanense* KCTC 42782^T^ was used in this study and routinely grown in the same media as strain D7^T^.

### 2.2. DNA Extraction, Purification and Sequencing 

Genomic DNA was extracted using an X-DNA purification kit (Xtrem Biotech, Granada, Spain) from an overnight culture of strain D7^T^ in R2A medium. The 16S rRNA gene was amplified by PCR using the universal primers for bacteria 16F27 and 16R1488 [46]. The obtained PCR product was then purified, cloned into the pGEM-T vector (Promega, Madison, WI, USA), and sequenced by direct sequencing using the ABI prism dye-terminator, cycle-sequencing ready-reaction kit and the ABI prism 377 sequencer according to PerkinElmer’s instructions. The GenBank/EMBL/DDBJ accession number for the sequence of the 16S rRNA gene is MG773714. The genome of strain D7^T^ was sequenced by using the Illumina MiSeq methodology (PE 150 *×* 2).

### 2.3. Phylogenetic Analysis Based on 16S rRNA Gene Sequence Comparison

Phylogenetic analyses based on the 16S rRNA gene were conducted as described previously [38,47]. The phylogenetic neighbors’ identification and the pairwise 16S rRNA gene sequence similarities calculations were carried out by using the EzBiocloud server “www.ezbiocloud.net” [48] (accessed on April 2021). Phylogenetic and molecular evolutionary analyses were conducted using Mega v. 7 [49]. Clustering was determined using the neighbor-joining and maximum-likelihood algorithms and the evolutionary distances were computed using the Jukes–Cantor method [50]. The analysis involved 34 nucleotide sequences and the stability of the clusters was determined by a bootstrap analysis (1000 replications).

### 2.4. Genomic Analyses

Illumina reads of strain D7^T^ were trimmed using a combination of software tools implemented in the BBMap project [51], and de novo assembled using SPADES v3.11.1 [52]. CheckM v1.0.18 [53] and Quast v5.0.2 [54] were used for assembly quality checks. The genome of strain D7^T^ was deposited in GenBank/EMBL/DDBJ under the accession number JAHREP000000000.

The genome of strain D7^T^ was annotated using BlastKOALA [55] and metabolic pathways were analyzed using KEGG [56]. 

Average Nucleotide Identity (ANI) and in silico DNA–DNA hybridization (DDH) values were calculated using the OrthoANI-usearch (OrthoANIu) software [57] and the Genome-to-Genome Distance Calculator (GGDC) website [58] with formula 2 [59], respectively.

InteractiVenn software [60] was used to display the Venn diagram.

### 2.5. Phylogenomic Reconstruction

Predicted protein sequences were compared using an all-versus-all BLAST search [61]. A total of 134 proteins were shared between all studied genomes and aligned using MUSCLE v3.8.31 [62]. The concatenated and aligned orthologous genes were used to build the phylogenomic tree in Mega v. 7 [49]. 

### 2.6. Phenotypic Characterization

Phenotypic analysis was conducted in order to characterize strain D7^T^. The optimum and the range of salt growth conditions for strain D7^T^ were evaluated in R2A medium to which were added different NaCl concentrations: 0, 0.5, 1, 3, 5, 7.5, 10, 15, 20, 25 and 30% (*w*/*v*). The optimal (and range) growth pH of D7^T^ was evaluated by growing the strain under different pH values (4, 5, 6, 7, 8, 9, 10 and 11). These pH values were reached using the following buffer systems: 0.1 M citric acid/0.1 M sodium citrate (pH 4.0–5.0); 0.1 M KH_2_PO_4_/0.1 M NaOH (pH 6.0–8.0); 0.1 M NaHCO_3_/0.1 M Na_2_CO_3_ (pH 9.0–10.0); and 0.2 M KH_2_PO_4_/0.1 M NaOH (pH 11.0) [63]. The effect of different temperatures (0, 5, 15, 20, 25, 28, 30, 32, 35, 37, 40 and 45 °C) was also assessed using marine broth (MB; Difco, Sparks, MD, USA). 

Gram staining was performed according to the method described by Komagata [64]. Growth under anaerobic conditions was determined in an anaerobic jar using AnaeroGen (Oxoid) and an anaerobic indicator (Oxoid, Hampshire, UK) using marine agar (MA; Difco). Motility was observed using log-phase culture according to the hanging-drop method [65]. Oxidase activity was determined with 1% (*v*/*v*) tetramethyl-p-phenylenediamine [66] and catalase activity was examined by bubble production with 3% (*v*/*v*) H_2_O_2_ solution [65]. The reduction of nitrate and nitrite and gas production were detected by adding the Griess-Ilosvay’s reagent (Merck) in cultures grown in peptone broth supplemented with 1% KNO_3_. Other biochemical character, carbon utilization, sugar fermentation and enzymatic tests were carried out by using the GEN III MicroPlate^TM^ system (Biolog), API 20NE, API 50CH and API ZYM strips (bioMerieux, Marcy I’Etoile, France) according to the manufacturers’ instructions.

Scanning electron microscope images of strain D7^T^ were produced on an FIB-FESEM (CrossBeam NVision 40, Carl Zeiss SMT) to determine bacterium size and type of flagella.

### 2.7. Chemotaxonomic Characterization

The fatty acids of strain D7^T^ were analyzed at the Spanish Type Culture Collection (CECT). Cells were grown on MA for 48 h, incubated at 30 °C. The whole-cell composition of the fatty acids was determined by GC using the midi microbial identification system [67]. The fatty acid profile was obtained with an Agilent 6850 gas chromatograph using the database TSBA6 145 [68]. 

Analysis of polar lipids and respiratory quinones of strain D7^T^ was carried out by the Identification Service of DSMZ, Braunschweig, Germany. Polar lipids were extracted following the protocol described by Bligh and Dyer [69]. Polar lipids were separated by two-dimensional silica-gel thin-layer chromatography (Macherey-Nagel Art. No. 818135) following the protocol described by Tindall et al. [70]. The two-stage method described by Tindall [71,72] was used to first extract respiratory lipoquinones followed by polar lipids.

## 3. Results and Discussion

### 3.1. Phylogenetic Analysis

During the course of the study of a soil sample from Rambla Salada (38°07′27.1″ N 1°07′01.4″ W), a hypersaline river located in Murcia (southeast of Spain), a novel strain designated D7^T^ was isolated and selected for further studies.

**Figure 1:**
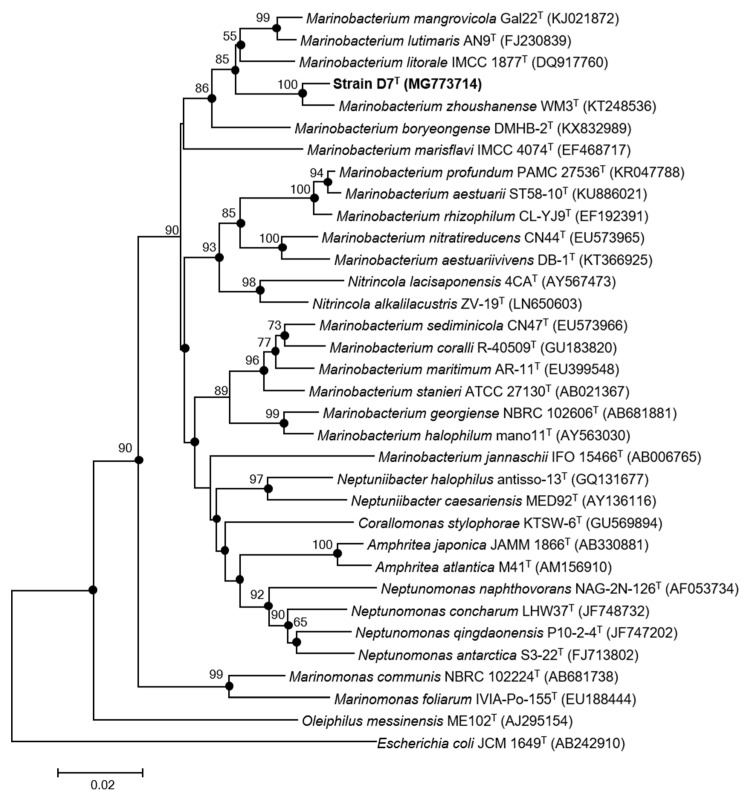
Neighbor-joining phylogenetic tree based on 16S rRNA gene sequences, showing the relationships of strain D7^T^ and related species. Sequences’ accession numbers are shown in parentheses. Bootstrap values based on 1000 replicates are listed as percentages at branching points; only values >50% are shown. Filled circles represent common nodes recovered in the maximum-likelihood algorithm representation. *Escherichia coli* JCM 1649^T^ was used as an outgroup. Bar, 0.02 substitutions per nucleotide position.

Based on the 16S rRNA gene sequence analysis, strain D7^T^ (1490 bp) was most closely related to the genus *Marinobacterium*, exhibiting the highest 16S rRNA gene sequence similarity to *Marinobacterium zhoushanense* WM3^T^ (98.0%), followed by *Marinobacterium lutimaris* DSM 22012^T^ (95.7%), *Marinobacterium mangrovicola* Gal22^T^ (95.3%) and *Marinobacterium litorale* IMCC 1877^T^ (95.1%). Moreover, the 16S rRNA gene sequence similarities to other genera, such as *Neptunomonas* and *Nitrincola*, were always equal or lower than 93.5%. The phylogenetic analysis based on the multiple sequence alignment of the 16S rRNA gene using the neighbor-joining algorithm (Figure 1), indicated that strain D7^T^ belongs to the genus *Marinobacterium* clustering with *Marinobacterium zhoushanense* WM3^T^ but was located in an independent branch with a high bootstrap value (Figure 1), indicating that the new strain could represent a new member of the genus *Marinobacterium*. A phylogenetic tree devised using the maximum-likelihood algorithm exhibited similar topologies.

### 3.2. Genomic Characteristics

The draft genome sequence of strain D7^T^ was obtained and compared with that of the closest phylogenetic species, *Marinobacterium zhoushanense* CGMCC 1.15341^T^, and with those of other members of the genus *Marinobacterium* with available genomes (Table 1). The draft genome of strain D7^T^ was de novo assembled into a total of 69 contigs, with a N50 value of 150,886 bp, a sequencing depth of 844X and a completeness of 99.9%. This genome sequence is in accordance with the minimal standards for the use of genome data for the taxonomy of prokaryotes [73]. The G+C content and genome size of strain D7^T^ were 59.2 mol% and 4,897,523 bp, respectively; those values were within the range of the genomes of the genus *Marinobacterium*, which ranged from 54.9 to 62.1 mol%, and from 3,653,655 to 5,637,742 bp, respectively (Table 1). Additional genomic characteristics are detailed in Table 1. Besides, the 16S rRNA gene sequence of strain D7^T^ obtained from the draft genome sequence was identical to that from the PCR, verifying the authenticity of this genome.

**Figure 2:**
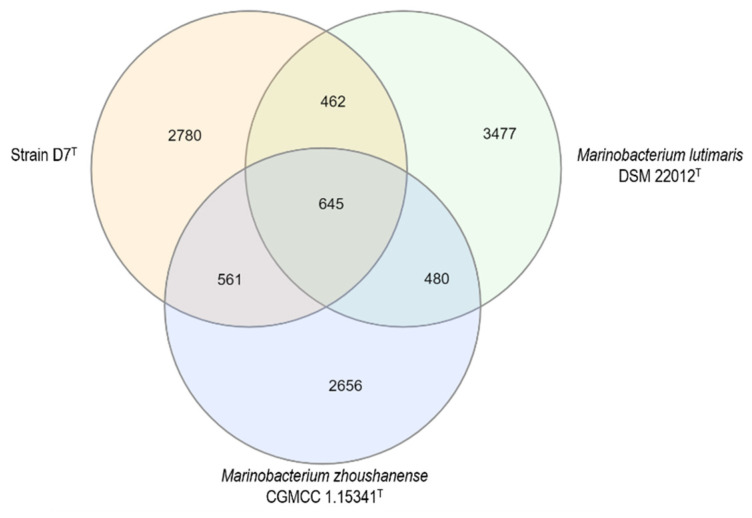
Venn diagram showing the number of genes shared between the genome of strain D7^T^, *Marinobacterium zhoushanense* CGMCC 1.15341^T^ and *Marinobacterium lutimaris* DSM 22012^T^.

In addition, a Venn diagram displaying the number of genes shared between strain D7^T^, *Marinobacterium zhoushanense* CGMCC 1.15341^T^ and *Marinobacterium lutimaris* DSM 22012^T^ was obtained (Figure 2). A total of 645 genes were shared between *M. zhoushanense* CGMCC 1.15341^T^, *M. lutimaris* DSM 22012^T^ and strain D7^T^, while 462 genes were shared between *M. lutimaris* DSM 22012^T^ and strain D7^T^, and 561 genes between *M. zhoushanense* CGMCC 1.15341^T^ and strain D7^T^ (Figure 2). A total of 2780 genes were identified as unique to strain D7^T^ (Figure 2). These results indicate that strain D7^T^ was unique from its closely related species.

### 3.3. Phylogenomic Analysis

**Figure 3:**
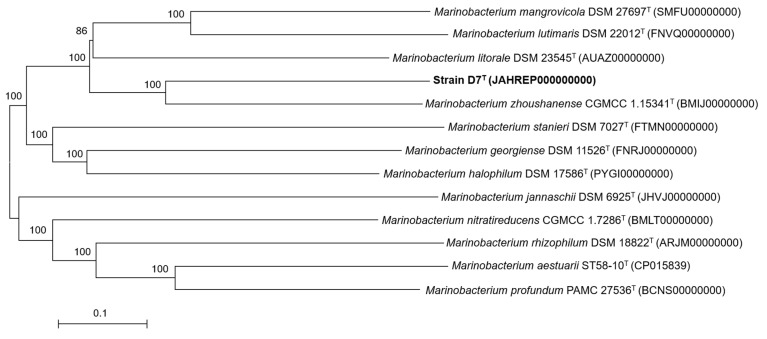
Neighbor-joining core gene phylogenetic tree including available genomes of the genus *Marinobacterium* and strain D7^T^. The tree was recovered from the alignment of 134 single-copy orthologous translated genes shared between these genomes. Bar, 0.1 substitutions per amino acid position.

According to the minimal standards for the use of genome data for the taxonomy of prokaryotes [73] and to confirm the phylogenomic relationships previously obtained by 16S rRNA gene sequence comparison, a phylogenomic tree based on core orthologous translated genes from strain D7^T^ and closely related members of the genus *Marinobacterium* was also obtained. A total of 134 single-copy orthologous genes were shared between all studied genomes and the phylogenomic tree reconstruction (Figure 3), clearly reflects that strain D7^T^ constitutes a monophyletic clade distinct from any other previously described species of the genus *Marinobacterium*, and therefore, supporting the placement of strain D7^T^ as a new species within this genus.

### 3.4. Average Nucleotide Identity (ANI) and In Silico DNA–DNA Hybridization (DDH)

To elucidate if strain D7^T^ may constitute a new species within the genus *Marinobacterium*, the genome-based sequence similarity analysis (ANI and in silico DDH) between strain D7^T^ and members of this genus was performed. For species delineation, the proposed and accepted boundaries for ANI and DDH are 95*–*96% and 70%, respectively [58,74,75]. 

**Figure 4:**
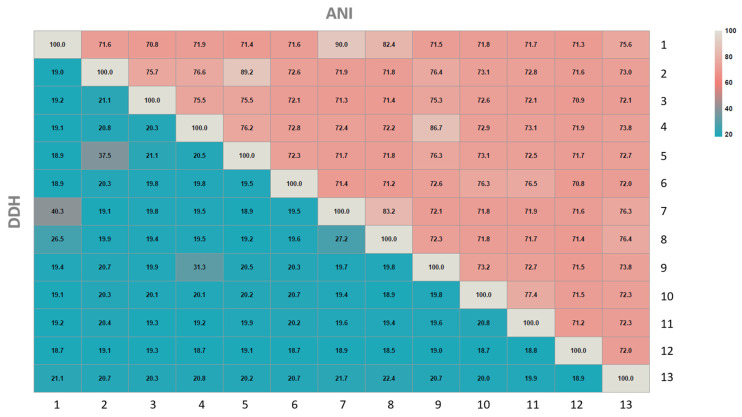
Heatmap representing the Average Nucleotide Identity (ANI) and in silico DNA–DNA hybridization (DDH) percentages between strain D7^T^ and related species of the genus *Marinobacterium* in which values are displayed by the colour key histograms on the upper panel. Strains: 1, *Marinobacterium profundum* PAMC 27536^T^; 2, *Marinobacterium lutimaris* DSM 22012^T^; 3, *Marinobacterium litorale* DSM 23545^T^; 4, Strain D7^T^; 5, *Marinobacterium mangrovicola* DSM 27697^T^; 6, *Marinobacterium georgiense* DSM 11526^T^; 7, *Marinobacterium aestuarii* ST58-10^T^; 8, *Marinobacterium rhizophilum* DSM 18822^T^; 9, *Marinobacterium zhoushanense* CGMCC 1.15341^T^; 10, *Marinobacterium stanieri* DSM 7027^T^; 11, *Marinobacterium halophilum* DSM 17586^T^; 12, *Marinobacterium jannaschii* DSM 6925^T^; 13, *Marinobacterium nitratireducens* CGMCC 1.7286^T^. Genome accession numbers are indicated in Table 1.

The ANI and DDH values between strain D7^T^ and *Marinobacterium zhoushanense* CGMCC 1.15341^T^, the closest phylogenetic neighbor, were 86.7% and 31.3%, respectively (Figure 4). In addition, the ANI and DDH estimations of strain D7^T^ in comparison to those of the other members of the genus *Marinobacterium* with available genomes were in all cases lower than the established cutoff values (Figure 4). These results support the conclusion that strain D7^T^ represents a novel species of the genus *Marinobacterium*. 

### 3.5. Chemotaxonomic Characterization

To taxonomically describe strain D7^T^ as a new species, the complete chemotaxonomic characterization of this strain was performed. The polar lipid profile of strain D7^T^ includes phosphatidylethanolamine (PE), phosphatidylglycerol (PG) and two uncharacterized lipids (L) (Figure S1). 

The fatty acids composition of strain D7^T^ and the type strains of related species of the genus *Marinobacterium* are shown in Table 2. In accordance with members of the genus *Marinobacterium* [3], the major cellular fatty acids (>10%) of strain D7^T^ were C_16:0_ (28.7%), summed feature 3 (C_16:1_ *ω*7*c*/C_16:1_ *ω*6*c*) (26.6%) and summed feature 8 (C_18:1_ *ω*7*c*/C_18:1_ *ω*6*c*) (25.2%). The fatty acid profile of strain D7^T^ was similar to those of the reference strains, but differing in their proportion (Table 2); hence, reaffirming its condition as a different species. 

The respiratory quinone of strain D7^T^ was ubiquinone-8 (Q-8), which is consistent with the rest of the members of the genus *Marinobacterium* [3].

### 3.6. Phenotypic Characterization

The phenotypic characteristics of strain D7^T^ were described and compared with those of *Marinobacterium zhoushanense* KCTC 42782^T^. Cells were Gram-staining-negative, short motile rods (0.5–0.6 × 1.0–1.8 µm) with a single polar flagellum (Figure S2). They were catalase and oxidase positive. Nitrate and nitrite were reduced. Glucose was not fermented. Weak growth was observed under anaerobic conditions. When tested on MA, growth of strain D7^T^ was observed at 15–40 °C (optimum, 37 °C), pH 5–9 (optimum, 7) and 0–7.5% (*w*/*v*) NaCl (optimum, 3%). Other characteristics of strain D7^T^ are given in the species description and those that differ from the strain type of the closest related species of the genus *Marinobacterium* are shown in Table 3.

### 3.7. Metabolism of Strain D7^T^

**Figure 5:**
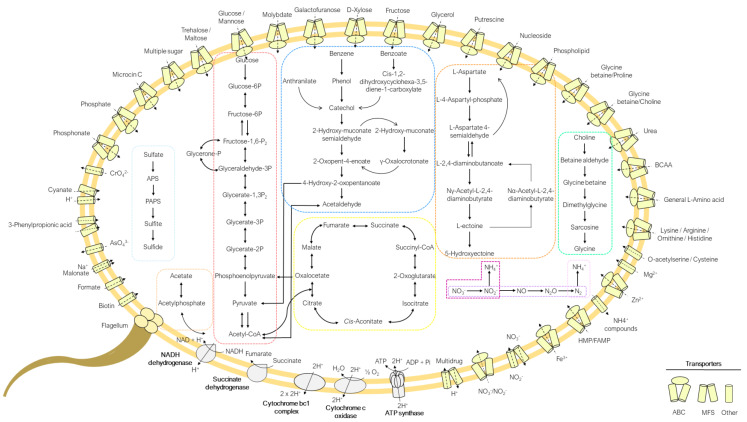
Metabolic reconstruction of strain D7^T^. Pathways included in color boxes from left to right: assimilatory sulfate reduction, acetate pathway, glycolysis, aromatic hydrocarbons’ degradation pathways, tricarboxilic acid cycle (TCA), ectoine biosynthesis and degradation pathways, nitrogen metabolism (dissimilatory nitrate reduction, denitrification and nitrogen fixation pathways), and glycine betaine biosynthesis and degradation pathways.

Metabolic insights after the in-depth genomic analysis of strain D7^T^ suggest a heterotrophic and chemolithotrophic lifestyle for this strain. In relation to its heterotrophic capabilities, central carbohydrate pathways such as glycolysis, gluconeogenesis, pentose phosphate, Entner–Doudoroff, tricarboxylic acid and glyoxylate cycle were detected (Figure 5). For pyruvate oxidation to acetyl-CoA, genes encoding pyruvate dehydrogenase (aerobic route) and pyruvate ferredoxin oxidoreductase (anaerobic route) were present. On the other side, a large number of ABC transporters for carbohydrate uptake (i.e., multiple sugar, glucose/mannose or fructose) were also identified in the studied genome (Figure 5). 

As part of the nitrogen metabolism of strain D7^T^, complete pathways for nitrogen fixation, dissimilatory nitrate reduction and denitrification were detected on its genome (Figure 5). While genomic evidence for nitrogen fixation and nitrate reduction to ammonia via dissimilatory nitrate reduction pathway has been previously suggested for other *Marinobacterium* species [3], the whole set of genes encoding all steps of the denitrification was not identified before in any other members of this group [3]. Only *Marinobacterium jannaschii* exhibited the almost complete route, but lacked the key enzyme (NirK) [3]. Moreover, in the specific case of strain D7^T^, the nitrate reduction capacity of this strain was also detected during the nitrate reduction phenotypic test. Several transporters for exogenous nitrogen-rich organic compounds uptake, such as Amt for ammonia, ABC for amino acids, putrescine or urea, with others, were found (Figure 5). In addition, various complete aminoacidic biosynthetic routes (i.e., serine, threonine, cysteine, valine/isoleucine, leucine, lysine, ornithine, arginine, proline, histidine and tryptophan) were encountered in this genome.

Like many halotolerant microorganisms, to cope with osmotically varying conditions, D7^T^ encode genes for the synthesis and uptake of different compatible solutes. Glycine betaine is one of the most important osmoprotectants in prokaryotes that could also serve as an energy and carbon source in hypersaline environments [76]. The key enzymes choline dehydrogenase (BetA) and glycine betaine aldehyde dehydrogenase (BetB), involved in the biosynthesis of glycine betaine from choline, were recognized during the genomic analysis of strain D7^T^ (Figure 5). The gene clusters *gbcAB*, *dgcAB* and *soxBDAG* for its further catabolism to glycine were also identified. This strain also possesses transporters for the uptake of this compound, such as the glycine betaine BCCT family and ABC transporters (Figure 5). No evidence for the alternative route for glycine betaine biosynthesis from glycine (via glycine and sarcosine methyltransferase and dimethylglycine methyltransferase) was identified in the studied genome.

Ectoine is another osmotic solute widely synthetized by halophilic bacteria [76]. The presence of the complete biosynthesis pathway in this strain suggests its capacity to additionally synthetize ectoine as a compatible solute (Figure 5). Besides, the ectoine hydroxylase (EctD) enzyme and the *doeBDAC* gene cluster, coding for Nα-acetyl-L-2,4-diaminobutyrate deacetylase (DoeB), diaminobutyrate transaminase (DoeD), ectoine hydrolase (DoeA) and aspartate semialdehyde dehydrogenase (DoeC), were also identified in this genome, indicating the ability to likewise synthetize its hydroxylated derivate, 5-hydroxyectoine, and to degrade ectoine, respectively (Figure 5). Considering ectoine biosynthesis is energetically more expensive than the betaine one, it would be reasonable to believe that this strain only synthetizes ectoine under starving betaine or choline concentrations. 

In addition, other several transporters related to osmotic stress for potassium uptake and sodium extrusion were also found in the genome.

Entire pathways for the catabolism of aromatic hydrocarbons (such as benzoate, benzene and anthranilate) to catechol were encountered in the genome of strain D7^T^, fueling the catechol *meta*-cleavage pathway for its further breaking down (Figure 5). In the same way, other *Marinobacterium* representatives (i.e., *M. aestuarii*, *M. stanieri*, *M. profundum* and/or *M. jannaschii*) were predicted to degrade benzoate or benzene, although in some cases through the *ortho*-cleavage pathway [3,16].

Noteworthily, strain D7^T^ encodes a sulfide:quinone oxidoreductase (SQR) which is predicted to oxidize sulfide to elemental sulfur, and thus exhibits a potential chemolithotrophic energetic metabolism for this strain. The SQR enzyme was also previously identified in the genomes of *M. jannaschii* and *M. litorale* [3]. Several others sulfur-based lithotrophy genes were also encoded by diverse *Marinobacterium* species [3]. On the other hand, the assimilatory sulfate reduction pathway via 3′-phosphoadenosine-5′-phosphosulfate (PAPS) was identified in the genome of strain D7^T^ (Figure 5), reflecting its capacity to reduce sulfate to sulfide with the aim of satisfying sulfur nutritional requirements.

Acetate is a major product of the metabolism formed during fermentation by many bacteria and other organisms [77]. In strain D7^T^, acetate is produced from Acetyl-CoA catalyzed by the phosphate acetyltransferase (Pta) and acetate kinase (AckA) enzymes (Figure 5). In addition to acetate, this reaction generates ATP, hence contributing to the energy metabolism of the cell.

Finally, consistently with its microscopical visualization, genes encoding flagellum were found during the genomic analysis. 

## 4. Conclusions

As a conclusion of the polyphasic taxogenomic analyses performed in this study, strain D7^T^ represents a new species within the genus *Marinobacterium*, for which the name *Marinobacterium ramblicola* sp. nov. is proposed. We enclose below the taxonomic description of this new species. The detailed genomic analysis of *Marinobacterium ramblicola* D7^T^ inferred a versatile energetic metabolism for this new taxon, characterized by a typical aerobic electron transport chain, oxidation of sulfur compounds, and nitrogen assimilation and fixation pathways. Likewise, based on the genomic inspection, it was suggested that the new species has the ability to biosynthetize or catabolize compatible solutes (i.e., ectoine or glycine betaine) and to degrade several aromatic hydrocarbons (i.e., benzene, benzoate and anthranilate). 

### Description of Marinobacterium ramblicola sp. nov.

*Marinobacterium ramblicola* [ram.bli’co.la. Spanish fem. n. *rambla* sandy ground; L. suff. -cola (from L. masc. or fem. n. *incola*) inhabitant; N.L. n. *ramblicola* inhabitant of a rambla].

Cells are motile Gram-stain-negative rods (0.5–0.6 × 1.0–1.8 µm). Colonies are 0.5 mm in size, pale yellow, circular, convex and opaque when grown on MA medium. Catalase and oxidase positive. Growth occurs at 15–40 °C (optimum, 37 °C), at pH 5–9 (optimum, 7) and with 0–7.5% (*w*/*v*) NaCl (optimum, 3%). Facultatively anaerobic. Cells are positive for nitrate reduction without gas formation. Indol is not produced. Gelatin and urea are not hydrolyzed. In Biolog GEN III MicroPlates^TM^, cells are positive for the assimilation of L-alanine, D-aspartic acid, L-aspartic acid, dextrin, D-maltose, D-trehalose, D-cellobiose, gentiobiose, sucrose, L-histidine, L-arginine, D-turanose, α-D-glucose, D-fructose, glycerol, glycyl-L-proline, L-glutamic acid, L-pyroglutamic acid, L-serine, pectin, D-galacturonic acid, L-galactonic acid lactone, acetic acid, acetoacetic acid, D-gluconic acid, quinic acid, p-hydroxy-phenylacetic acid, methyl pyruvate, D-lactic acid methyl ester, L-lactic acid, citric acid, α-hydroxy-butyric acid, α-keto glutaric acid, α-keto butyric acid, L-malic acid, β-hydroxy-D,L-butyric acid, bromo-succinic acid, Tween 40, γ-amino butyric acid, propionic acid, formic acid and sodium butyrate. Other organic substrates included in Biolog GEN III microplates are not utilized. Acids are produced from D-maltose and D-melezitose, but not from adonitol, D-arabinose, arbutin, D-arabitol, L-arabitol, D-cellobiose, D-fructose, D-glucose, glycerol, D-galactose, dulcitol, erythritol, L-lactose, D-mannose, D-ribose, D-xylose, L-xylose, methyl-β-D-xylopyranoside, L-sorbose, L-rhamnose, inositol, D-mannitol, D-sorbitol, methyl- α-D-glucopyranoside, methyl-α-D-mannopyranoside, N-acetylglucosamine, amygdalin, inulin, salicin, D-mellibiose, D-sucrose, D-raffinose, D-lyxose, starch, glycogen, D-trehalose, xylitol, gentiobiose, D-turanose, D-tagatose, D-fucose, L-fucose, potassium gluconate, potassium 2-ketogluconate or potassium 5-ketogluconate.

Enzymatic activities such as acid phosphatase, alkaline phosphatase, valine arylamidase, leucine arylamidase and napthol-AS-BI-phosphohydrolase are positive, but the activities cystine arylamidase, α-chymotrypsin, esterase (C4), esterase lipase (C8), lipase (C14), α-glucosidase, β-glucosidase trypsin, α-galactosidase, β-galactosidase, β-glucuronidase, *N*-acetyl- β-glucosaminidase, α-mannosidase and α-fucosidase are negative.

The isoprenoid quinone is Q-8. The polar lipids profile consists of phosphatidylethanolamine, phosphatidylglycerol and two uncharacterized lipids. Major fatty acids are C_16:0_, summed feature 3 (C_16:1_ *ω*7*c*/C_16:1_ *ω*6*c*) and summed feature 8 (C_18:1_ *ω*7*c*/C_18:1_ *ω*6*c*).

The type strain is D7^T^ (= CECT 9818^T^ = LMG 31312^T^), isolated from a hypersaline river located in Murcia, Spain.

The GenBank/EMBL/DDBJ accession numbers for the 16S rRNA gene sequence and for the draft genome are MG773714 and JAHREP000000000, respectively.

## Figures and Tables

**Table 1:** General features of the genomes of strain D7^T^ and other species of the genus *Marinobacterium*.

Feature	1	2	3	4	5	6	7	8	9	10	11	12	13
Size (bp)	4,897,523	4,734,355	5,191,608	3,925,261	3,653,655	5,174,280	4,378,172	5,568,333	4,979,947	5,546,883	5,637,742	5,360,582	4,680,330
Contigs	69	29	1	51	47	47	68	21	15	39	226	68	24
G+C (mol%)	59.2	58.4	58.8	54.9	56.0	55.2	56.4	57.5	57.1	62.1	57.2	58.5	55.6
N50 (bp)	150,886	548,647	5,191,608	167,945	130,350	201,845	137,897	869,882	922,765	457,680	61,011	143,318	388,460
Total genes	4522	4408	4614	3742	3442	4705	4252	5136	4517	4778	5051	4768	4380
Protein coding genes	4427	4285	4461	3625	3326	4569	4114	5024	4414	4635	4863	4604	4259
rRNA	3	7	18	8	3	8	9	3	ND	3	5	6	6
tRNA	59	73	83	56	71	62	57	69	68	70	82	66	73
Accession number	JAHREP000000000	BMIJ00000000	CP015839	FNRJ00000000	PYGI00000000	JHVJ00000000	AUAZ00000000	FNVQ00000000	SMFU00000000	BMLT00000000	BCNS00000000	ARJM00000000	FTMN00000000

Strains: 1, Strain D7^T^; 2, *Marinobacterium zhoushanense* CGMCC 1.15341^T^; 3, *Marinobacterium aestuarii* ST58-10^T^; 4, *Marinobacterium georgiense* DSM 11526^T^; 5, *Marinobacterium halophilum* DSM 17586^T^; 6, *Marinobacterium jannaschii* DSM 6925^T^; 7, *Marinobacterium litorale* DSM 23545^T^; 8, *Marinobacterium lutimaris* DSM 22012^T^; 9, *Marinobacterium mangrovicola* DSM 27697^T^; 10, *Marinobacterium nitratireducens* CGMCC 1.7286^T^; 11, *Marinobacterium profundum* PAMC 27536^T^; 12, *Marinobacterium rhizophilum* DSM 18822^T^; 13, *Marinobacterium stanieri* DSM 7027^T^.

**Table 2:** Fatty acid profile of strain D7^T^ and closely related species of the genus *Marinobacterium*.

Fatty Acids	1	2 ^a^	3 ^a^
**Saturated:**			
C_10:0_	0.7	-	-
C_12:0_	**5.9**	**5.3**	3.2
C_14:0_	0.8	-	-
C_16:0_	**28.7**	**22.0**	**22.8**
C_18:0_	0.3	TR	1.3
**Hydroxy:**			
C_10:0_ 3-OH	**6.7**	**5.8**	**6.4**
C_12:0_ 2-OH	**-**	-	1.7
C_16:0_ 3-OH	0.3	-	-
**Cyclo:**			
C_17:0_ cyclo	4.9	3.1	TR
**Summed features:**			
3 (C_16:1_ *ω*7*c*/C_16:1_ *ω*6*c*)	**26.6**	**29.5**	**16.9**
8 (C_18:1_ *ω*7*c*/C_18:1_ *ω*6*c*)	**25.2**	**31.8**	**45.0**

Strains: 1, Strain D7^T^; 2, *Marinobacterium zhoushanense* KCTC 42782^T^; 3, *Marinobacterium lutimaris* KACC 13703^T^. Cells of strain D7^T^, and strains *M. zhoushanense* KCTC 42782^T^ and *Marinobacterium lutimaris* KACC 13703^T^, were grown on marine agar at 30 °C and 25 °C, respectively, for 48 h. The major fatty acids (>5% of the total fatty acids) are highlighted in bold. -, not detected; TR, trace amount (<1%). Data represent percentages of total fatty acids. ^a^ Data from Kang et al. [12].

**Table 3:** Differential phenotypic features of strain D7^T^ in comparison to those of the closest related species of the genus *Marinobacterium*.

Characteristic	1	2
Cell size (µm)	0.5–0.6 × 1.0–1.8	0.4–0.6 × 1.0–2.0 ^a^
Temperature range for growth (optimum) (°C)	15–40 (37)	15–43 (37–40) ^a^
pH range for growth (optimum)	5–9 (7)	5.5–9.5 (6.5–7.5) ^a^
NaCl range concentration for growth (optimum) (%, *w*/*v*)	0–7.5 (3)	0.25–9 (1–1.5) ^a^
Nitrate reduction	+	-
Citrate utilization	-	+
**Carbon source utilization in Biolog GEN III:**		
D-Cellobiose	+	-
Gentiobiose	+	-
D-Serine	-	+
D-Mannitol	-	+
D-Arabitol	-	+
D-Aspartic Acid	+	-
D-Glucuronic Acid	-	+
Mucic Acid	-	+
D-Saccharid Acid	-	+
Citric Acid	+	-
Sodium Butyrate	+	-
**Enzymatic activities in API ZYM:**		
α-Glucosidase	-	+
**Acid production in API 50CH:**		
D-Maltose	+	-
D-Sucrose	-	+
D-Trehalose	-	+

Strains: 1, Strain D7^T^; 2, *Marinobacterium zhoushanense* KCTC 42782^T^. All data from this study, except ^a^ that were obtained from Han et al. [15]. +, positive; -, negative. Other features included in the species description were common for both strains.

## Supplementary Materials

The following are available online at https://www.mdpi.com/article/10.3390/microorganisms9081654/s1, Figure S1: Two-dimensional TLC of polar lipids from strain D7T; Figure S2: Electron micrographs of negatively stained preparation of cells of strain D7^T^.
