# Supplementary material for: Taxogenomic and Metabolic Insights into Marinobacterium ramblicola sp. nov., a New Slightly Halophilic Bacterium Isolated from Rambla Salada, Murcia
Source: Microorganisms. 2021 Aug 3;9(8):1654. doi: 10.3390/microorganisms9081654 (PMC8398569; doi:10.3390/microorganisms9081654)
Supplement: Supplementary file 1 [file microorganisms-09-01654-s001.zip › microorganisms-1298359-SI.pdf]

microorganisms

MDPI

Supplementary Material

Taxogenomic and Metabolic Insights *Marinobacterium ramblicola* sp. nov., a New Slightly Halophilic Bacterium Isolated from Rambla Salada, Murcia

Ana Durán-Viseras [1,2*], David J. Castro[1,2], José Carlos Reina[1,2], Victoria Béjar[1,2] and Fernando Martínez-Checa[1,2*]

1 Microbial Exopolysaccharide Research Group, Department of Microbiology, Pharmacy Faculty, Campus de Cartuja s.n., 18071 Granada, Spain; anaduran@us.es (A.D.-V.), castricoxpc@gmail.com (D.J.C), josecreina@ugr.es (J.C.R), vbejar@ugr.es (V.B.), fmcheca@ugr.es (F.M.-C.)
2 Biomedical Research Center, Institute of Biotechnology, 18016 Granada, Spain
* Correspondence: anaduran@us.es fmcheca@ugr.es

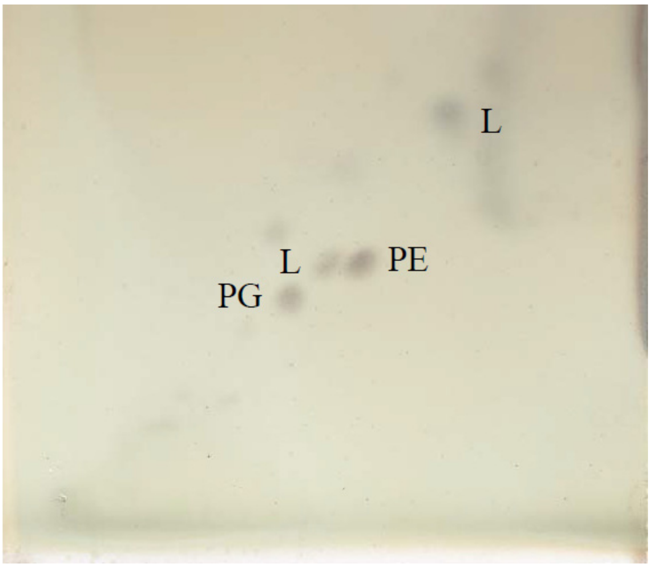

Figure S1. Two-dimensional TLC of polar lipids from strain D7^T. **Abbreviations:** PE, phosphatidylethanolamine; PG, phosphatidylglycerol; L, lipid.

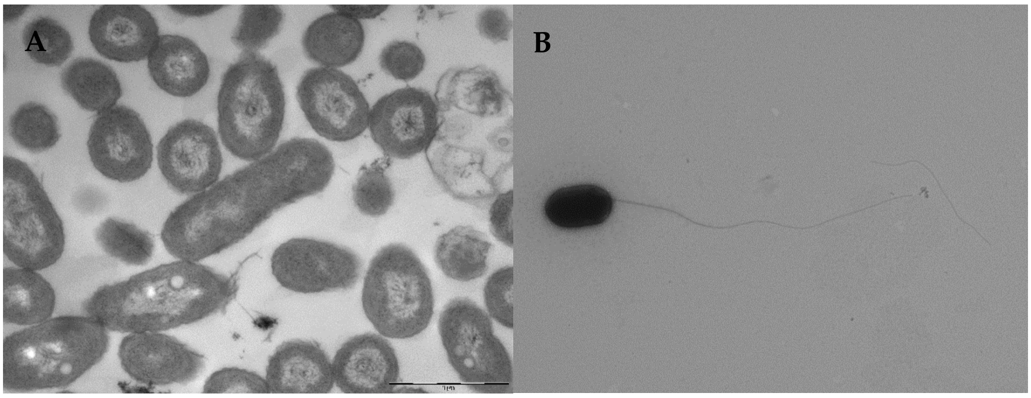

Figure S2. Electron micrographs of negatively stained preparation of cells of strain D7^T. Cells were grown on MA plates at 32 °C for 24 h with shaking at 150 rpm and stained with % (w/v) uranyl acetate (Bar, 1 μm).

Copyright: © 2021 by the authors. Submitted for possible open access publication under the terms and conditions of the Creative Commons Attribution (CC BY) license (http://creativecommons.org/licenses/by/4.0/).
